# Cdc25A inhibits autophagy-mediated ferroptosis by upregulating ErbB2 through PKM2 dephosphorylation in cervical cancer cells

**DOI:** 10.1038/s41419-021-04342-y

**Published:** 2021-11-06

**Authors:** Chen Wang, Jie Zeng, Li-Jie Li, Min Xue, Si-Li He

**Affiliations:** 1 Department of Gynecology and Obstetrics, the Third Xiangya Hospital of Central South University, 410013 Changsha, Hunan Province P.R. China; 2 Pharmacy Intravenous Admixture Services, the Third Xiangya Hospital of Central South University, 410013 Changsha, Hunan Province P.R. China

**Keywords:** Cervical cancer, Cervical cancer

## Abstract

Cervical cancer is the leading cause of cancer-related deaths in women, and treatment for cervical cancer is very limited. Emerging evidence suggests that targeting ferroptosis is a promising way to treat cancer. Here, we investigated the role of ferroptosis in cervical cancer, with a focus on the Cdc25A/PKM2/ErbB2 axis. Cervical cancer cells were treated with sorafenib to induce ferroptosis. Cellular MDA/ROS/GSH/iron detection assays were used to measure ferroptosis. MTT assays were performed to assess cell viability. qRT-PCR, western blot, and immunostaining assays were performed to measure the levels of proteins. Autophagy was monitored by fluorescence microscopy. Nuclear and cytosolic fractions were isolated to examine the location of PKM2 modifications. Co-IP experiments were conducted to determine the Cdc25A/PKM2 interaction. ChIP assays were performed to measure the binding affinity between H3K9Ac and the ErbB3 promoter, and a dual luciferase assay was performed to examine the transcriptional activity of ErbB2. A nude mouse xenograft model was used to examine the effects of the Cdc25A/ErbB2 axis on tumour growth in vivo. Cdc25A was elevated in human cervical cancer tissues but was reduced during sorafenib-induced ferroptosis of cervical cancer cells. Overexpression of Cdc25A inhibited sorafenib-induced ferroptosis by dephosphorylating nuclear PKM2 and suppressing autophagy. Cdc25A regulated autophagy-induced ferroptosis by increasing ErbB2 levels via the PKM2–pH3T11–H3K9Ac pathway. Cdc25A increased the resistance of cervical cancer to sorafenib, while knockdown of ErbB2 blocked these effects. Cdc25A suppressed autophagy-dependent ferroptosis in cervical cancer cells by upregulating ErbB2 levels through the dephosphorylation of PKM2. These studies revealed that Cdc25A/PKM2/ErbB2 pathway-regulated ferroptosis could serve as a therapeutic target in cervical cancer.

## Introduction

Cervical cancer is one of the most common causes of cancer-related deaths in women, affecting millions of women worldwide [1]. In underdeveloped countries or areas, it is the leading cause of death in women. It is primarily caused by persistent infections of the human papilloma virus (HPV) [2]. The incidence and death rates have dropped due to the increased use of early tests. However, for patients with regional or distant metastasis, the treatments are limited, and the prognosis remains dismal [3]. Therefore, it is necessary to explore novel treatments for cervical cancer, which requires a better understanding of the pathogenesis and molecular mechanisms of cancer development and progression.

Ferroptosis is a newly discovered type of programmed cell death characterized by an iron-dependent accumulation of lipid peroxidation [4, 5]. Ferroptosis-inducing factors such as system Xc- or glutathione peroxidase 4 inhibitors regulate glutathione peroxidase and decrease antioxidant capacity, leading to the accumulation of lipid reactive oxygen species (ROS) and eventually oxidative cell death [5]. Compounds including erastin and sorafenib have been shown to induce ferroptosis by inhibiting system Xc- [6]. Ferroptosis is largely associated with autophagy, a conserved process that helps maintain cell homoeostasis by degrading dysfunctional organelles and proteins in autophagosomes [4, 7]. Activation of autophagy causes the degradation of ferritin, the major intracellular protein that stores iron, resulting in an increase in intracellular iron and ferroptosis. Autophagy machinery, including NCOA4, BECN1, and STAT3, greatly contributes to ferroptotic cell death by inhibiting system Xc- and activating lipophagy and ferritinophagy [8]. Furthermore, blockade of autophagy abrogates the accumulation of cellular ROS and labile iron, thereby suppressing ferroptosis [9]. Emerging evidence shows that ferroptosis plays an important role in physiological processes and diverse diseases, including cancers [10, 11]. Aberrant ferroptosis has been observed in a variety of cancers, and ferroptosis inhibitors or activators can greatly affect cancer progression [11]. However, the function of ferroptosis in cervical cancer is largely unknown.

The cell division cycle 25 (Cdc25) family is a class of phosphatases that have critical roles in regulating the cell cycle [12]. Cdc25A is an isoform that primarily regulates the G1-S transition. Aberrant expression of Cdc25A has been observed in many cancers [13]. For instance, Cdc25A was upregulated in triple-negative breast cancer (TNBC), and a Cdc25A inhibitor suppressed TNBC growth. Additionally, overexpression of Cdc25A is associated with a poor prognosis of human hepatocellular carcinoma [14]. Recently, it has been reported that Cdc25A is elevated in cervical cancer tissues and cells [15]. Furthermore, upregulation of Cdc25A increases the resistance of cancer cells to radiotherapy, while knockdown of Cdc25A promotes cervical cancer cell apoptosis [15]. Nevertheless, the exact role of Cdc25A in cervical cancer and the signalling pathways involved are not fully understood. ErbB2 is a member of the epidermal growth factor receptor (EGFP)/ErbB family composed of four highly homologous receptor tyrosine kinases (ErbB1–4) [16]. Previous studies show that inhibition of ErbB2 induces apoptosis of cervical cancer cells [17]. In addition, ErbB2 regulates autophagy by acting on Beclin-1 [18]. However, the exact functional role of ErbB2 in cervical cancer remains elusive. Pyruvate kinase M2 (PKM2) is a terminal enzyme in the glycolytic pathway and has been implicated in various cancers [19]. PKM2 has many phosphorylation sites, including Ser37 and Tyr105, and its distinct phosphorylation has different effects on PKM2 function [20]. For instance, Tyr105 phosphorylation inhibits PKM2 activity, while Ser37 phosphorylation promotes PKM2 translocation to the nucleus to serve as a transcriptional coactivator [20]. Whether this posttranslational modification is involved in cervical cancer is unknown.

In the present study, we sought to examine the role of ferroptosis in cervical cancer, with a focus on the Cdc25A/ErbB2 axis. We found that Cdc25A was decreased during sorafenib-induced ferroptosis in cervical cancer cells. Overexpression of Cdc25A suppressed sorafenib-induced ferroptosis. Mechanistically, we showed that Cdc25A dephosphorylated PKM2 at Ser37 in the nucleus and inhibited autophagy. In addition, Cdc25A upregulated ErbB2 levels through the PKM2–pH3T11–H3K9Ac pathway. Furthermore, overexpression of Cdc25A enhanced the resistance of cervical cancer cells to sorafenib-induced ferroptosis, and the knockdown of ErbB2 blocked the effects of Cdc25A. Together, the results of our study demonstrate that Cdc25A protects cervical cancer cells against autophagy-mediated ferroptosis by upregulating ErbB2 levels through PKM2 dephosphorylation. This work sheds light on the mechanisms underlying cervical cancer cell ferroptosis, providing avenues for the development of improved therapeutic strategies for cervical cancer.

## Materials and methods

### Human cervical cancer specimens

Human cervical cancer specimens (*N* = 24) were taken from cervical cancer patients during surgery at the Third Xiangya Hospital of Central South University. The non-tumour tissues near the cancers were collected simultaneously from the same patients. Patients did not receive preoperative treatments. All patients consented to the study. The study was reviewed and received approval from the Ethics Committee of the Third Xiangya Hospital of Central South University. All specimens were placed in liquid nitrogen immediately after collection and then stored in a freezer (−80 °C).

### Cell culture and treatment

Human cervical cancer cell lines (SiHa and CaSki) were used and purchased from the Chinese Academy of Sciences Cell Bank (Shanghai, China). The medium used for cell culture was composed of Dulbecco’s modified Eagle’s medium (DMEM) (Gibco, CA, USA) plus 10% foetal bovine serum (Thermo-Fisher Scientific, MA, USA) and 1% penicillin-streptomycin (Gibco, USA). The cells were maintained in a CO_2_ incubator at 37 °C.

For drug treatments, cultured cells were grown to 70–80% confluence and then treated with various concentrations of the ferroptosis inducer sorafenib (0, 0.5, 1, 2.5, 5, 10 μM), autophagy inhibitor 3-MA (10 mM), ferrostatin-1 (Fer1, 1 μM) or iron-chelating agent (deferoxamine mesylate, DFO, 100 μM) for 48 h as indicated in the figures before harvest for further analysis.

### Plasmids and cell transfection

The full-length sequences of Cdc25A, p-PKM2 ser37, and PKM2S37D phosphorylation-mimic mutants were cloned into the overexpression construct (pcDNA3.1). Sh-ErbB2 and control sh-NC were synthesized by GenePharma (Shanghai, China). Lipofectamine 3000 (Invitrogen, China) was utilized as the reagent for cell transfection. Briefly, cells were cultured to 70–80% confluence and then added to the plasmid together with Lipofectamine 3000 at a ratio of 1:1.

### Subcellular fractionation

Cell nucleus and cytosolic fractions were isolated as previously described [21]. Briefly, cells were homogenized with sucrose buffer (320 mM sucrose) and centrifuged at 800 × *g* for 10 min at 4 °C to obtain the nuclear pellet. The supernatant was further centrifuged at 20,000 × *g* for 20 min at 4 °C to yield the membrane fraction (pellet) and a cytosolic fraction (supernatant). Cytosolic fraction (supernatant) was saved for further analysis. Lysis buffer was added to resuspend the nucleus followed by sonication for protein extraction and further analysis.

### 3-(4,5-dimethylthiazol-2-yl)-2,5-diphenyltetrazolium bromide (MTT) assay

Cell viability was measured with a MTT assay (Sigma). Cultured transfected cells were seeded in a 96-well plate (Corning Life Sciences, USA) and treated with drugs at the indicated concentrations. MTT (5 mg/mL) was added at 24, 48, 72, and 96 h post-treatment and incubated with cells for 4 h. Afterwards, the supernatant was removed, and 150 µL dimethyl sulfoxide was added to the plate. The absorbance at 490 nm was measured using a plate reader (Glomax; Promega, MI, USA).

### Lipid peroxidation assay (malondialdehyde (MDA))

Cellular MDA levels were determined by the lipid peroxidation assay kit (Abcam, ab118970, UK) according to the manufacturer’s protocol. Cultured or transfected cells following drug treatments were harvested in MDA lysis solution. Thiobarbituric acid (TBA) solution was mixed with standards or test samples, and the mixture was incubated for 1 h at 95 °C followed by ice-cooling for 10 min. The absorbance of the TBA–MDA adduct at 532 nm was measured with a plate reader (GloMax; Promega, MI, USA).

### Cellular ROS detection assay

ROS levels were determined with a standard ROS detection kit (Abcam, ab113851, UK) according to the manufacturer’s protocol. Cultured or transfected cells following drug treatments were switched to fresh media and then added to ROS/Superoxide Detection Mix for incubation in the cell incubator for 1 h. The media was discarded, and the cells were washed with 1 × washing buffer. A coverslip was used to overlay the cells. The fluorescence intensity (Ex/Em = 490/525 nm) was analysed using confocal microscopy.

### Cellular glutathione (GSH) detection assay

Intracellular GSH levels were measured with a standard GSH detection assay kit (Abcam, ab112132, UK) according to the manufacturer’s instructions. Cultured or transfected cells following drug treatments were incubated with Thiol Green Dye for 30 min at 37 °C and centrifuged at 1000 rpm for 5 min to pellet the cells. The cells were resuspended in a culture medium, and the fluorescence intensity was analysed with flow cytometry.

### Cellular iron detection assay

Intracellular iron levels (Fe^2+^) were determined with a standard iron assay kit (Abcam, ab83366, UK) according to the manufacturer’s protocol. Cultured or transfected cells following drug treatments were washed with cold PBS and then lysed in iron assay buffer followed by centrifugation at 16,000 × *g* for 10 min to collect the supernatants. An iron reducer was added to both the samples and standards and incubated at 37 °C for 30 min. Afterwards, an iron probe was added and incubated for an additional 60 min at 37 °C in the dark. Output (OD 593 nm) was measured on the microplate reader.

### RNA extraction and qRT-PCR

TRIzol (Invitrogen, China) was used to extract total RNA from cervical cancer cells according to the manufacturer’s instructions. DNaseI was included in the lysis buffer to avoid DNA contamination. A commercial kit (cDNA synthesis kits, Thermo-Fisher, China) was utilized to generate cDNAs through reverse transcription. SYBR Green Master Mix (Invitrogen, China) was used for quantitative PCR. Relative mRNA expression levels of Cdc25A and ErbB2 were normalized to GAPDH mRNA as an internal control. The relative expression was calculated by the 2^−ΔΔCt^ method. The primers listed as follows were from GenePharma (Shanghai, China):

Cdc25A forward primer: 5′-TGTGCCGGTATGTGAGAGAG-3′;

Cdc25A reverse primer: 5′-TGCGGAACTTCTTCAGGTCT-3′;

ErbB2 forward primer: 5′-AAAGGCCCAAGACTCTCTCC-3’′;

ErbB2 reverse primer: 5′-CTCTGGGTTCTCTGCCGTAG-5′;

GAPDH forward primer: 5′-CCAGGTGGTCTCCTCTGA-3′;

GAPDH reverse primer: 5′-GCTGTAGCCAAATCGTTGT-3′.

### Western blot analysis

Proteins from cervical cancer cells or tumours were extracted by utilizing RIPA lysis buffer (Abcam, China) according to a standard protocol. A DC Protein Assay Kit (Bio-Rad, China) was utilized to quantify the protein concentrations. Equal amounts of protein from each sample were loaded into SDS-polyacrylamide gels and separated through electrophoresis. Later, proteins in the gels were transferred to PVDF membranes (Sigma-Aldrich, China). BSA (3%) was used to block the membranes for 30–60 min at room temperature, specific primary antibodies were added, and the mixture was incubated at 4 °C overnight. The antibodies were discarded, and TBST was utilized to wash the membranes three times before incubation with specific secondary antibodies for 1–2 h at room temperature. Protein band intensities were detected by using a standard ECL kit. The primary antibodies used in the study were as follows: anti-p62 antibody (1: 1000, Cell Signalling Technology, #5114T, USA); anti-LC3-I antibody (1: 1000, Millipore, ABC929, USA); anti-LC3-II antibody (1: 1000, Millipore, ABC929, USA); anti-ErbB2 (1: 1000, Cell Signalling Technology, #2242S, USA); anti-p-PKM2 Ser37 (1: 1000, ThermoFisher, #PA5-37684, USA); anti-PKM2 (1: 1000, Cell Signalling Technology, #3198S, USA); anti-Cdc25A (1: 1000, ThermoFisher, #PA5-77092, USA); anti-p-H3T11 (1: 1000, Abcam, ab5168, UK); anti-H3K9Ac (1: 1000, Cell Signalling Technology, #9671, USA); anti-FTH1 (1: 1000, Abcam, ab65080, UK); anti-TfR1 (1: 1000, Abcam, ab84036, UK); and anti-Tubulin (1:2000, Abcam, ab6046, UK).

### Chromatin immunoprecipitation (ChIP) assay

ChIP was performed using a commercial ChIP kit (Cell Signalling Technology, USA) according to the manufacturer’s protocol. For each ChIP, 5 μg of anti-H3K9Ac and 1 μL of normal rabbit IgG were used. IgG antibody was included as a negative control. After immunoprecipitation, chromosomal DNA was purified. The ErbB2 promoter region was detected by using PCR.

### Immunofluorescence

Autophagy was monitored by fluorescence microscopy. Briefly, cells were transfected with the GFP-LC3 construct before drug treatment using Lipofectamine 3000 (Invitrogen, USA) according to the manufacturer’s protocol. At the end of the indicated experiments, cells were fixed in 4% paraformaldehyde (PFA) for 10–12 min at room temperature and then washed with PBS followed by confocal imaging.

### Co-immunoprecipitation (Co-IP)

Cervical cancer cells were transfected with Cdc25A and flag-PKM2 for 48 h. Nuclear and cytosolic fractions were isolated as described above and then lysed with lysis buffer supplemented with protease inhibitor. Protein concentrations were quantified with a Pierce BCA protein assay (Thermo Fisher Scientific, USA). Equal amounts of proteins were incubated with Anti-Flag antibody (Thermo Fisher, USA) for 1 h at 4 °C and then with Protein-A Sepharose beads (Abcam, UK) overnight at 4 °C. The beads were then washed in lysis buffer followed by elution with SDS loading buffer. The elution proceeded with standard western blotting.

### Immunohistochemistry (IHC)

The cervical tissues were fixed in 4% PFA at 4 °C overnight, washed with PBS, and then embedded in paraffin. The tumour tissues were sliced into 10-μm-thick sections and dried overnight at 37 °C on glass slides. The dried slices were deparaffinized in xylene first followed by rehydration through a graded concentration of alcohol. Hydrogen peroxide (3%) was used to quench the sections, and 5% bovine serum albumin (BSA) was added to block the slices for 1 h at room temperature. Primary antibody (anti-Cdc25A; 1:500; ThermoFisher, #PA5-77092, USA) was added and incubated with the slices at 4 °C overnight. The next day, the antibody was washed off with PBS, and secondary antibodies were added and incubated with sections for 2 h at room temperature. Substrates of the Envision system-HRP from the kit (Dako REAL Ebision kit; DAKO, Glostrup, Denmark) were added to incubate with stained slices as the manufacturer’s protocol described, and the signals were analysed with a light microscope.

### Dual-luciferase assay

Fragments of the cDNAs containing the promoter region of ErbB2 were amplified by PCR and cloned into the Sall and BamHl restriction sites of the pGLO luciferase reporter vector (Promega, WI, USA). Cervical cancer cells were seeded in individual wells of 24-well plates first overnight, and then recombinant constructs were transfected into cancer cells by using Lipofectamine 3000. The co-transfected cells were treated with sorafenib for 48 and then harvested to measure relative luciferase activities by the Luciferase Reporter Gene Assay Kit (Dual-Glo^TM^ Luciferase Assay System, Promega, Madison, WI, USA).

### Nude mouse xenograft model

All animal experiments were reviewed and received approval by the Animal Care and Use Committee of the Third Xiangya Hospital of Central South University. Four-week-old male nude mice (*N* = 72) were purchased from SJA Laboratory Animal Co., Ltd. (Hunan, China), and raised in the standard animal facility room. Cervical cancer cells were infected with the control vector, Cdc25A, Cdc25A + sh-NC, and Cdc25A + sh-ErbB2 lentiviruses for 24 h and then unilateral subaxillary subcutaneously injected into nude mice (1 × 10^7^ cells per mouse) to induce tumours. Non-infected cervical cancer cells were used for the saline group and sorafenib group. For sorafenib treatment, the mice received intraperitoneal injection of sorafenib (10 mg/kg) every 2 days starting at day 8. Saline was used as a control, and the mice in the saline group were intraperitoneally injected with saline every 2 days starting at day 8. Tumours were monitored on a daily basis for 30 days. Tumour length (*L)* and width (*W*) were analysed to quantify the tumour volume (*V*): *V* (mm^3^) = 0.5 × (*W*)^2^ × (*L*). Finally, tumours were dissected to measure weight, and the tumour tissues were harvested for further analysis.

### Quantifications and statistical analysis

All experimental data were analysed in GraphPad Prism 7. Statistical analyses included unpaired Student’s *t*-test (for two groups) and one-way ANOVA (for more than two groups) and are indicated in the figure legends. The data are presented as the mean ± standard deviation (SD).

## Results

### Cdc25A was elevated in human cervical cancer tissues

**Fig. 1:**
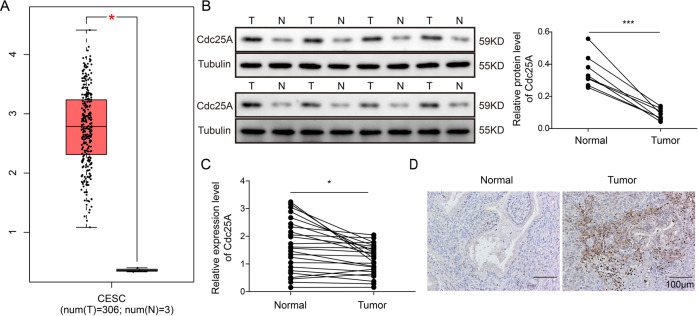
Cdc25A was elevated in human cervical cancer tissues. **A** Cdc25A levels in cervical cancer tissues and normal cervical tissues from the TCGA dataset. **B** Cdc25A protein levels in human cervical cancer tissues and adjacent non-tumour tissues from eight cervical cancer patients. **C** Cdc25A mRNA levels in human cervical cancer tissues and adjacent non-tumour tissues from cervical cancer patients (*N* = 24). **D** IHC analysis of Cdc25A levels in human cervical cancer tissues and adjacent non-tumour tissues from cervical cancer patients. Each experiment was repeated three times. *P < 0.05; ***P < 0.001.

To study the function of Cdc25A in cervical cancer, we first measured its expression level in cervical cancer tissues. By analysing the TCGA dataset, we found that the level of Cdc25A in cervical cancer tissues was much higher than that in normal cervical tissues (Fig. 1A). We also collected human cervical cancer specimens and adjacent non-tumour tissues from patients diagnosed with cervical cancer. Consistently, both the protein and mRNA levels of Cdc25A were significantly upregulated in cervical cancer tissues compared to non-tumour tissues (Fig. 1B, C). Furthermore, using IHC, we observed that the signal intensity of Cdc25A was much higher in cervical cancer tissues than in non-tumour cervical tissues (Fig. 1D). Therefore, we concluded that Cdc25A was elevated in cervical cancer tissues.

### Cdc25A was reduced during sorafenib-induced ferroptosis of human cervical cancer cells

**Fig. 2:**
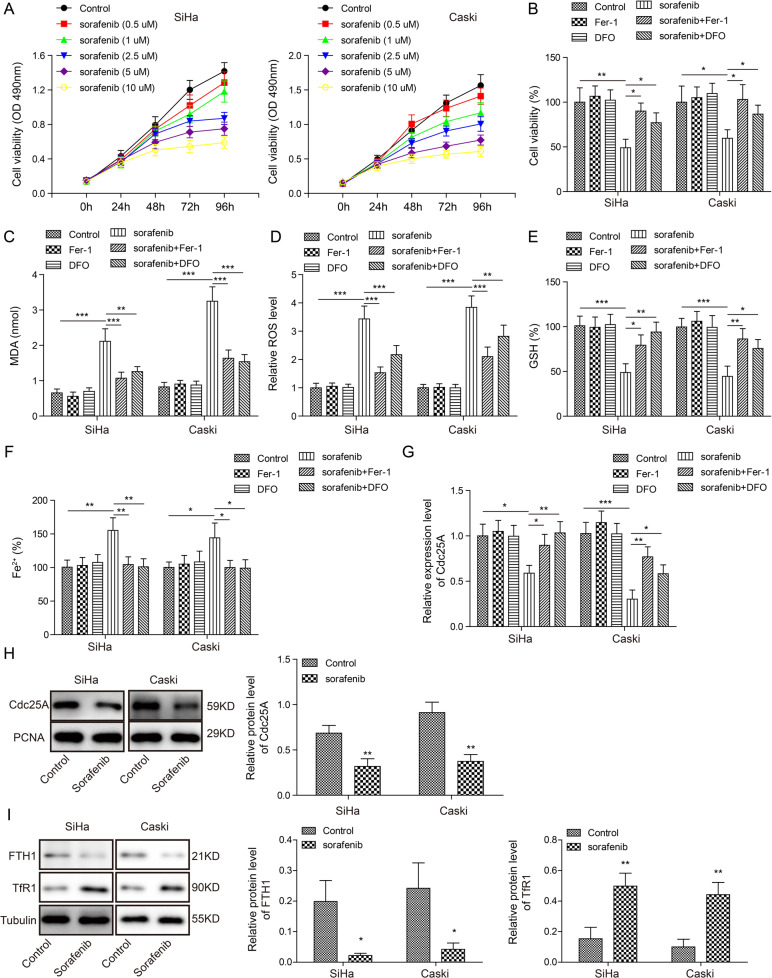
Cdc25A was reduced during sorafenib-induced ferroptosis of human cervical cancer cells. **A** The MTT assay to analyse cell viability following sorafenib treatment with different concentrations. **B** The MTT assay to analyse cell viability upon various drug treatments. **C** Cellular MDA levels after drug treatment. **D** Cellular ROS levels after 48 h of drug treatment. **E** Cellular GSH levels after 48 h of drug treatment. **F** Cellular iron levels after 48 h of drug treatment. **G** and **H** Cdc25A mRNA (**G**) and protien levels (**H**) in cervical cancer cells following 48 h of sorafenib treatment. **I** Protein levels of FTH1 and TfR1 in cervical cancer cells following 48 h of sorafenib treatment. Each experiment was repeated three times. *P < 0.05; **P < 0.01; ***P < 0.001.

As an anti-cancer drug, sorafenib has been shown to induce ferroptosis in cancer cells [22]. We treated human cervical cancer cells with various concentrations of sorafenib (0, 0.5, 1, 2.5, 5, 10 μM) to induce ferroptosis. Using the MTT assay, we observed that sorafenib treatment markedly decreased the viability of cancer cells, with larger decreases detected at higher concentrations (Fig. 2A). We used 10 μM sorafenib for further experiments since this concentration exhibited the largest effect. To test whether the decreased viability caused by sorafenib specifically resulted from activation of ferroptosis, we treated cancer cells with the ferroptosis inhibitor Fer1 or DFO together with sorafenib. As shown in Fig. 2B, Fer1 or DFO treatment alone did not affect the viability of cancer cells. However, the reduction in cell viability induced by sorafenib was suppressed by cotreatment with Fer1 or DFO (Fig. 2B). We measured cellular MDA/ROS/GSH/iron levels following drug treatment by using the corresponding kits and found that sorafenib treatment greatly increased MDA/ROS/iron levels but decreased GSH levels. Co-treatment with Fer1 and DFO reversed the changes induced by sorafenib, suggesting that ferroptosis occurred in sorafenib-treated cancer cells (Fig. 2C–F). To study the role of Cdc25A in this process, we measured its expression level. We found that Cdc25A mRNA and protein levels were both significantly reduced in sorafenib-treated cells (Fig. 2G, H). In addition, we observed a reduction in ferritin heavy chain (FTH1), which is responsible for intracellular iron storage, and an elevation in transferrin receptor 1 (TfR1) upon sorafenib treatment, which regulates iron influx (Fig. 2I). Together, these results showed that sorafenib induced ferroptosis in cervical cancer cells and downregulated Cdc25A expression levels.

### Overexpression of Cdc25A suppressed ferroptosis in cervical cancer cells

**Fig. 3:**
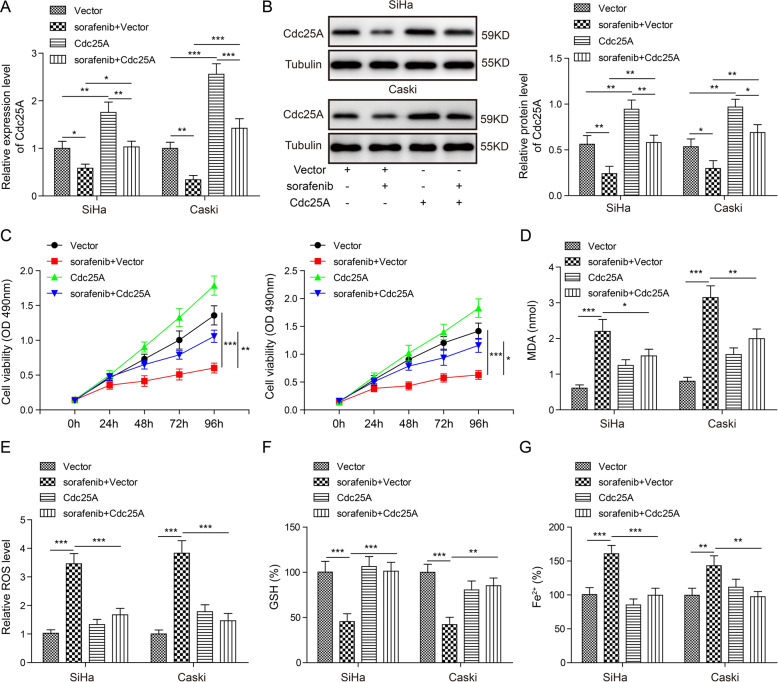
Overexpression of Cdc25A suppressed sorafenib-induced ferroptosis. **A** Relative Cdc25A mRNA levels in transfected cells with or without sorafenib treatment. **B** Relative Cdc25A protein levels in transfected cells with or without sorafenib treatment. **C** The MTT assay to analyse the viability of transfected cells following sorafenib treatment. **D** Cellular MDA levels in transfected cells after sorafenib treatment. **E** Cellular ROS levels in transfected cells after sorafenib treatment. **F** Cellular GSH levels in transfected cells after sorafenib treatment. **G** Cellular iron levels in transfected cells after sorafenib treatment. Each experiment was repeated three times. *P < 0.05; **P < 0.01; ***P < 0.001.

To investigate the function of Cdc25A in sorafenib-induced ferroptosis, we manipulated Cdc25A levels and tested the effects on ferroptosis. As expected, sorafenib treatment in vector-transfected cells drastically decreased Cdc25A levels (Fig. 3A, B). Overexpression of Cdc25A restored the Cdc25A level in sorafenib-treated cells (Fig. 3A, B). By performing the MTT assay, we found that overexpression of Cdc25A recovered the viability of cancer cells treated with sorafenib (Fig. 3C). In addition, intracellular MDA/ROS/GSH/iron levels in sorafenib-treated cells were all recovered by Cdc25A overexpression compared to vector-treated cells (Fig. 3D–G). Taken together, these data showed that overexpression of Cdc25A in cancer cells inhibited cervical cancer cell ferroptosis.

### Cdc25A dephosphorylated PKM2 to suppress sorafenib-induced ferroptosis

**Fig. 4:**
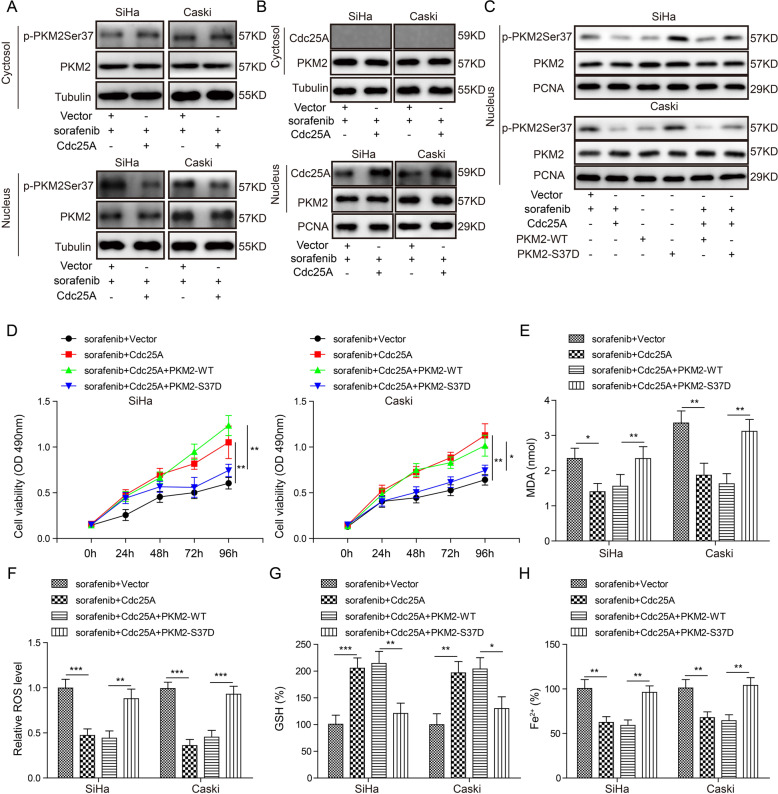
Cdc25A dephosphorylated PKM2 to suppress sorafenib-induced ferroptosis. **A** Relative p-PKM2 Ser37 levels in the nucleus and cytosol of transfected cells following sorafenib treatment. **B** Co-IP was used to analyse the interaction of Cdc25A and PKM2 in the nucleus and cytosol. **C** Relative p-PKM2 Ser37 levels in transfected cells with or without sorafenib treatment. **D** The MTT assay to analyse the viability of transfected cells following sorafenib treatment. **E** Cellular MDA levels in transfected cells after sorafenib treatment. **F** Cellular ROS levels in transfected cells after sorafenib treatment. **G** Cellular GSH levels in transfected cells after sorafenib treatment. **H** Cellular iron levels in transfected cells after sorafenib treatment. Each experiment was repeated three times. *P < 0.05; **P < 0.01; ***P < 0.001.

Next, we examined the mechanisms underlying the effects of Cdc25A. As a dual-specificity phosphatase, Cdc25A dephosphorylates downstream proteins. Previous studies have suggested that PKM2 can be dephosphorylated by Cdc25A at Ser37 and that this dephosphorylation event is involved in tumorigenesis [23]. We speculated that a similar mode of regulation occurred in cervical cancer. We isolated nuclear and cytosolic fractions and separately examined p-PKM2 Ser37 levels. We found that overexpression of Cdc25A in sorafenib-treated cells significantly decreased p-PKM2 Ser37 levels in the nucleus but not in the cytosol (Fig. 4A). Furthermore, immunoprecipitation of PKM2 greatly pulled down Cdc25A from the nucleus but not in the cytosol (Fig. 4B), suggesting that Cdc25A binds and dephosphorylates PKM2 in the nucleus. Co-expression of PKM2 S37D, a phosphorylation-mimic mutant, but not wild-type (WT) PKM2 restored the phosphorylation level of PKM2 in Cdc25A-transfected cells (Fig. 4C). Moreover, the viability of Cdc25A/PKM2 S37D co-transfected cervical cancer cells was decreased to a similar extent as vector-transfected cells by sorafenib treatment (Fig. 4D). Overexpression of Cdc25A together with PKM2-WT still recovered cell viability following sorafenib treatment compared to that of the vector-transfected cells, which showed the same effects as that of the Cdc25A-overexpressing group (Fig. 4D). Consistently, co-expression of PKM2 S37D but not PKM2-WT reversed the effects of Cdc25A overexpression on cellular MDA/ROS/GSH/iron levels in ferroptotic cell death (Fig. 4E–H). Therefore, we concluded that Cdc25A suppressed cervical cancer cell ferroptosis by dephosphorylating PKM2 in the nucleus.

### Cdc25A inhibited autophagy to suppress sorafenib-induced ferroptosis in cervical cancer cells

**Fig. 5:**
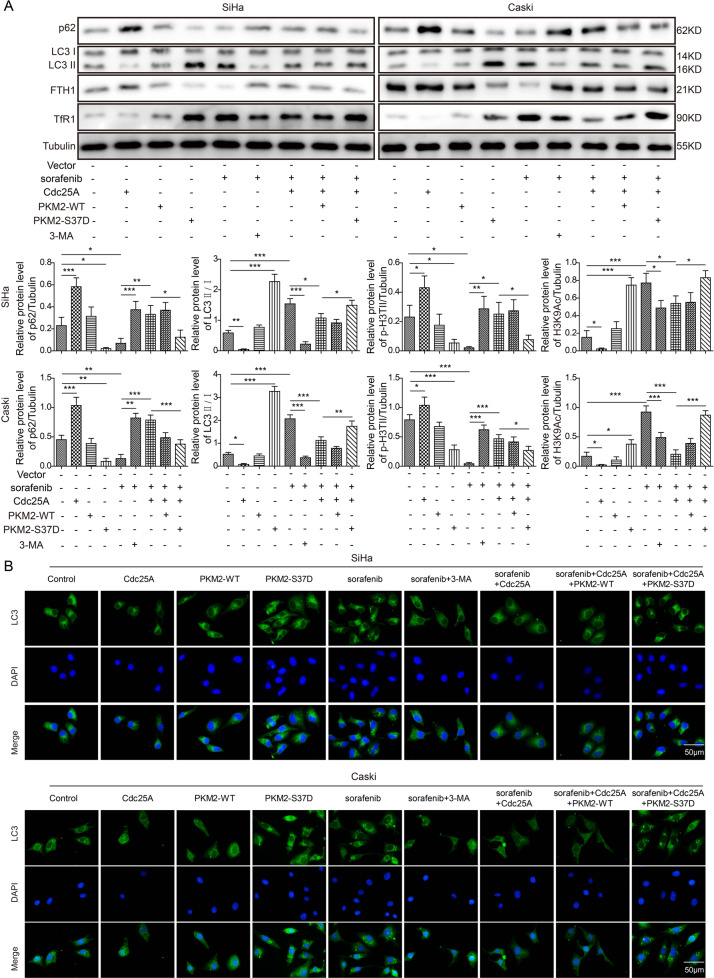
Cdc25A inhibited autophagy to suppress sorafenib-induced ferroptosis. **A** Relative protein levels of p62, LC3-I, LC3-II, FTH1, and TfR1 in transfected cells with or without sorafenib treatment. **B** Relative GFP-LC3 signalling in transfected cells with or without sorafenib treatment. Each experiment was repeated three times. *P < 0.05; **P < 0.01.

Ferroptosis is strongly related to autophagy [24]. We thus examined how autophagy was involved in ferroptosis in cervical cancer cells. As shown in Supplemental Fig. 1, sorafenib treatment increased cellular ROS and iron levels, while co-treatment with 3-MA, an autophagy inhibitor, and sorafenib suppressed these effects, supporting the notion that autophagy contributes to ferroptosis. By western blotting, we found that Cdc25A overexpression alone increased p62 and FTH1 levels but decreased the LC3-II/LC-I ratio and TfR1 level in cervical cancer cells, while PKM2-S37D overexpression showed the opposite effects (Fig. 5A), suggesting that autophagy is suppressed by Cdc25A but enhanced by PKM2 Ser37 phosphorylation. Transfection of PKM2-WT did not change their protein levels (Fig. 5A). Following sorafenib treatment, we observed reductions in p62 and FTH1 and increases in the LC3-II/LC-I ratio and TfR1 level (Fig. 5A), suggesting that sorafenib promotes autophagy. Co-treatment with 3-MA or overexpression of Cdc25A upregulated p62 and FTH1 levels and downregulated the LC3-II/LC-I ratio and TfR1 level in sorafenib-treated cells (Fig. 5A), indicating that overexpression of Cdc25A suppressed sorafenib-induced autophagy in cervical cancer cells. Furthermore, co-overexpression of PKM2-S37D but not PKM2-WT reversed the effects of Cdc25A overexpression in sorafenib-treated cells (Fig. 5A). Using immunostaining techniques along with the GFP-LC3 construct, we observed that the GFP-LC3 signal was significantly reduced in Cdc25A-transfected cells, while PKM2-S37D overexpression and sorafenib treatment increased the GFP-LC3 signal (Fig. 5B). Co-treatment with 3-MA or overexpression of Cdc25A suppressed the GFP-LC3 signal increase induced by sorafenib treatment (Fig. 5B). Again, co-overexpression of PKM2-S37D but not PKM2-WT reversed the effects of Cdc25A overexpression in sorafenib-treated cells (Fig. 5B). Taken together, these results showed that Cdc25A inhibited sorafenib-induced autophagy, which might be the mechanism underlying its role in cervical cancer cell ferroptosis.

### Cdc25A regulated ErbB2 via the PKM2–pH3T11–H3K9Ac pathway

**Fig. 6:**
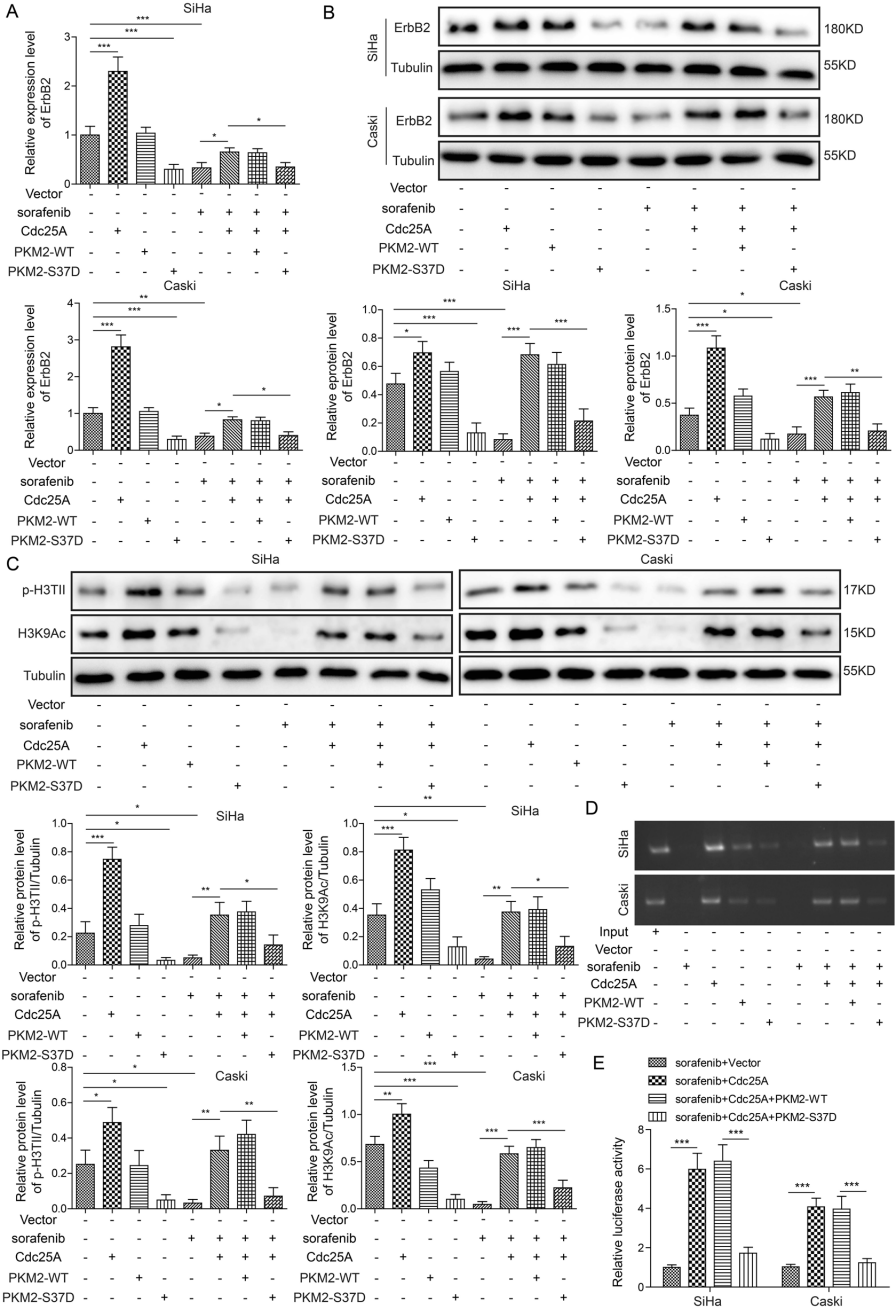
Cdc25A regulated ErbB2 via PKM2–pH3T11–H3K9Ac pathway. **A** Relative ErbB2 mRNA levels in transfected cells with or without sorafenib treatment. **B** Relative ErbB2 protein levels in transfected cells with or without sorafenib treatment. **C** Relative protein levels of p-H3T11 and H3K9Ac in transfected cells with or without sorafenib treatment. **D** ChIP analysis of the binding between H3K9Ac and the ErbB2 promoter. **E** Relative luciferase activities of ErbB2 in transfected cells following sorafenib treatment. Each experiment was repeated three times. *P < 0.05; **P < 0.01; ***P < 0.001.

ErbB2 is an EGFP and has been shown to be involved in autophagy [18]. Additionally, a recent study indicated that PKM2 promotes ErbB2 expression via pH3T11-acetylation of the histone H3 lysine 9 (H3K9Ac) pathway [25]. Therefore, we investigated the function of ErbB2 in cervical cancer cell ferroptosis. Overexpression of Cdc25A significantly increased ErbB2 mRNA and protein levels in cancer cells, while sorafenib treatment or PKM2-S37D overexpression reduced these levels (Fig. 6A, B). Moreover, Cdc25A overexpression restored ErbB2 levels in sorafenib-treated cancer cells (Fig. 6A, B). Overexpression of PKM2-S37D but not PKM2-WT suppressed Cdc25A-induced recovery in sorafenib-treated cancer cells (Fig. 6A, B). Furthermore, Cdc25A upregulated p-H3T11 and H3K9Ac levels, while PKM2-S37D and sorafenib treatment downregulated these levels (Fig. 6C). Cdc25A overexpression again recovered these levels in sorafenib-treated cells, and these recoveries were suppressed by co-overexpression of PKM2-S37D (Fig. 6C). By performing ChIP-qPCR experiments, we found that Cdc25A significantly increased the binding of H3K9Ac to the ErbB2 promoter, while sorafenib treatment or PKM2-S37D overexpression decreased the binding (Fig. 6D). Moreover, Cdc25A overexpression restored this binding in sorafenib-treated cancer cells (Fig. 6D). Overexpression of PKM2-S37D, but not PKM2-WT, suppressed the restoration mediated by Cdc25A in sorafenib-treated cells (Fig. 6D). We also performed a dual luciferase assay to examine the transcriptional activity of the ErbB2 promoter. Consistently, overexpression of Cdc25A substantially increased the relative luciferase activity of the ErbB2 promoter, while PKM2-S37D but not PKM2-WT reversed this regulation (Fig. 6E). Taken together, these results showed that Cdc25A increased ErbB2 levels by PKM2-mediated signalling.

### Cdc25A regulated cervical cancer cell ferroptosis via ErbB2

**Fig. 7:**
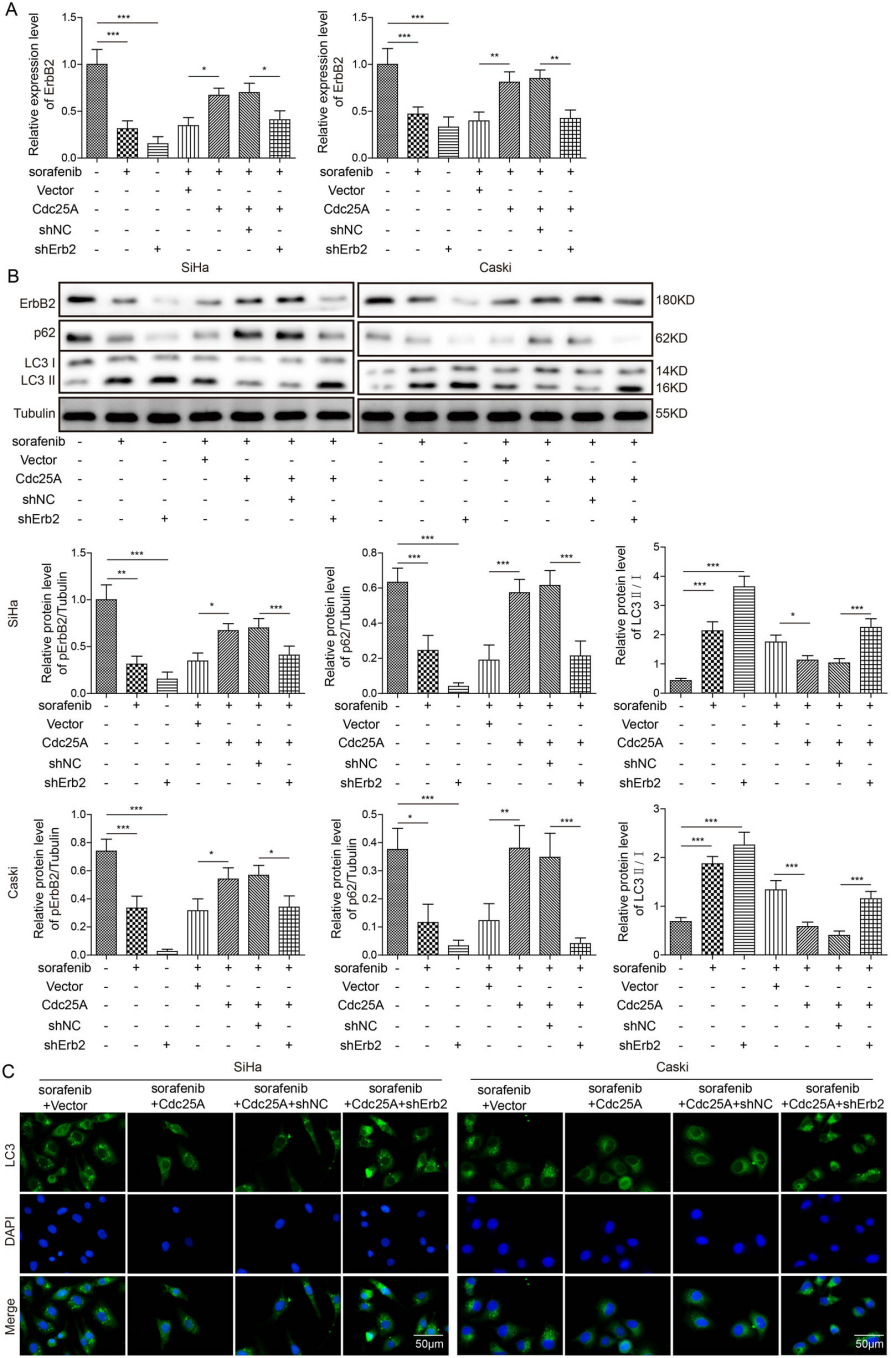

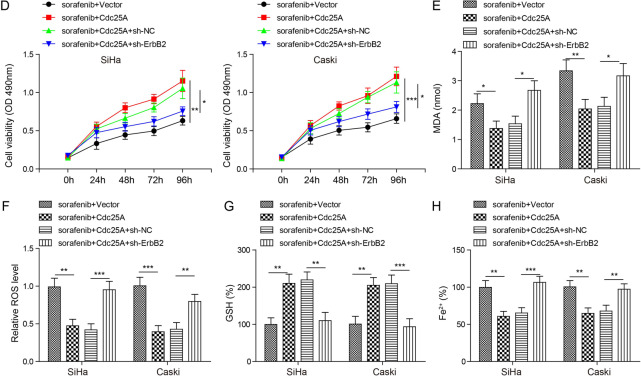
Cdc25A regulated sorafenib-induced ferroptosis via ErbB2. **A** Relative ErbB2 mRNA levels in transfected cells with or without sorafenib treatment. **B** Relative protein levels of ErbB2, p62, LC3-I, and LC3-II in transfected cells with or without sorafenib treatment. **C** Relative GFP-LC3 signalling in transfected cells following sorafenib treatment. **D** The MTT assay to analyse the viability of transfected cells following sorafenib treatment. **E** Cellular MDA levels in transfected cells after sorafenib treatment. **F** Cellular ROS levels in transfected cells after sorafenib treatment. **G** Cellular GSH levels in transfected cells after sorafenib treatment. **H** Cellular iron levels in transfected cells after sorafenib treatment. Each experiment was repeated three times. *P < 0.05; **P < 0.01; ***P < 0.001.

To directly test the role of ErbB2 in Cdc25A-induced ferroptosis resistance, we manipulated its expression level and examined the effects. As expected, sorafenib treatment or ErbB2 knockdown alone greatly diminished ErbB2 mRNA and protein levels and p62 levels but upregulated the LC3-II/LC3-I ratio (Fig. 7A, B). Overexpression of Cdc25A in sorafenib-treated cells increased ErbB2 mRNA and protein levels and p62 expression but decreased the LC3-II/LC3-I ratio (Fig. 7A, B). Co-transfection of sh-ErbB2 blocked these Cdc25A-mediated changes in sorafenib-treated cells (Fig. 7A, B). In addition, Cdc25A decreased the GFP-LC3 signal in sorafenib-treated cells, while co-transfection of sh-ErbB2 recovered the signal (Fig. 7C), indicating that knockdown of ErbB2 blocks Cdc25A-induced anti-autophagy. Furthermore, knockdown of ErbB2 in Cdc25A-transfected cells inhibited the increased viability mediated by Cdc25A overexpression in sorafenib-treated cancer cells (Fig. 7D). Similarly, intracellular levels of MDA/ROS/GSH/iron all returned to the level found in vector-transfected cells in Cdc25A- and sh-ErbB2-co-transfected cells (Fig. 7E–H). Therefore, these data provided strong evidence that Cdc25A suppressed sorafenib-induced autophagy and ferroptosis by increasing ErbB2.

### Cdc25A increased the resistance of cervical carcinoma to sorafenib in vivo

**Fig. 8:**
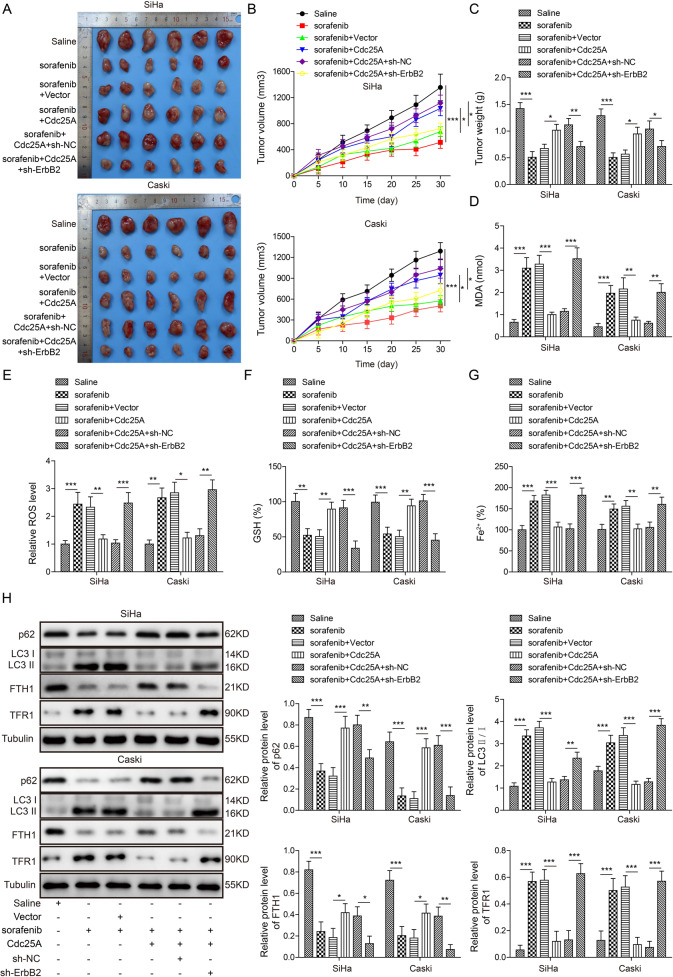
Cdc25A increased the resistance of cervical carcinoma to sorafenib in vivo. **A** Representative image of tumours from each group of mice. *N* = 6 in each group. **B** Relative tumour volume with time in each group of mice. **C** Tumour weight in each group of mice. **D** Cellular MDA levels in tumours from each group of mice. **E** Cellular ROS levels in tumours from each group of mice. **F** Cellular GSH levels in tumours from each group of mice. **G** Cellular iron levels in tumours from each group of mice. **H** Relative protein levels of p62, LC3-I, LC3-II, FTH1, and TFR1 in tumours from each group of mice. Each experiment was repeated three times. *P < 0.05; **P < 0.01; ***P < 0.001.

Finally, we evaluated the function of Cdc25A in cervical carcinoma in vivo by using a nude mouse xenograft model. We implanted non-transfected or transfected human cervical cancer cells (vector, Cdc25A, Cdc25A + sh-NC, Cdc25A + sh-ErbB2) into nude mice, and 7 days later, saline or sorafenib was injected intraperitoneally every other day for 30 days. As shown in Fig. 8A–C, the tumours in mice implanted with non-transfected cancer cells and injected with saline (saline) became progressively larger with time. Compared to those in saline-treated mice, the tumours in sorafenib-injected mice were consistently smaller and lighter (Fig. 8A–C), indicating that sorafenib suppresses cervical tumour growth in vivo. The tumours in the sorafenib-injected mice implanted with Cdc25A-transfected cells (sorafenib + Cdc25A) were consistently and significantly larger than those in sorafenib-injected mice bearing vector-transfected cells (sorafenib + vector) (Fig. 8A–C), suggesting that overexpression of Cdc25A inhibits the anti-tumour effect of sorafenib. However, the tumours in sorafenib-injected mice implanted with Cdc25A + sh-ErbB2-transfected cells were smaller than those in sorafenib-injected mice bearing Cdc25A alone (sorafenib + Cdc25A) or Cdc25A + sh-NC (sorafenib + Cdc25A + sh-NC)-transfected cells) (Fig. 8A–C), showing that the knockdown of ErbB2 blocks the effects of Cdc25A. At the end of the experiment, we sacrificed the animals and harvested the tumours for further analysis. We found that the levels of MDA/ROS/iron in the tumours were increased while GSH was diminished after sorafenib treatment (Fig. 8D–G). Overexpression of Cdc25A inhibited these changes, but co-overexpression of Cdc25A and sh-ErbB2 reversed the effects of Cdc25A overexpression (Fig. 8D–G). The western blotting results showed that sorafenib treatment decreased p62 and ferritin heavy chain 1 (FTH1) levels but increased the LC3-II/LC3-I ratio and transferrin receptor 1 (TFR1) expression, suggesting that sorafenib induces autophagy and ferroptosis (Fig. 8H). Overexpression of Cdc25A in cancer cells reversed these changes, while knockdown of ErbB2 blocked the effects of Cdc25A overexpression (Fig. 8H). These results demonstrated that Cdc25A increased the resistance of cervical tumours to the ferroptosis inducer sorafenib via ErbB2 in vivo.

## Discussion

Despite great advances made in diagnosis and research, cervical cancer remains the leading cause of cancer-related death in women. Finding a method to effectively kill cancer cells without affecting normal cells is a key challenge for cancer treatment, including cervical cancer treatment. Ferroptosis is a new type of regulated and programmed cell death, and ferroptosis inducers have exhibited promising results in killing cancer cells. Nevertheless, there is limited research on the role of ferroptosis in cervical cancer. Here, we used sorafenib, a ferroptosis inducer, to show that ferroptosis modulation could provide a way to kill cervical cancer cells. Furthermore, we reveal the molecular mechanisms that underlie the effects of sorafenib on cervical cancer. These results will help us understand the pathogenesis of cervical cancer and provide novel avenues for future therapy development.

Sorafenib is an FDA-approved multi-kinase inhibitor drug to treat a variety of cancers, such as kidney cancer and liver cancer [26–28]. Recently, accumulating studies have shown that sorafenib kills cancer cells by inducing ferroptosis. Here, we observed similar functions of sorafenib in cervical cancer cells. Sorafenib treatment substantially increased cellular iron/MDA/ROS levels but decreased GSH levels in cervical cancer cells, which are typical features of ferroptosis [29]. Interestingly, we found that Cdc25A could suppress sorafenib-induced ferroptosis in cervical cancer cells. As a dual-specificity protein phosphatase, Cdc25A plays a crucial role in regulating the cell cycle by acting in opposition to the cyclin-dependent kinases (CDKs) [12]. It has been shown that Cdc25A inhibits apoptosis [12], but whether it is involved in ferroptosis has remained elusive. We provide evidence that Cdc25A acts to suppress ferroptosis by restraining the autophagy process. There is a large amount of crosstalk between autophagy and ferroptosis, as they share many molecules and pathways [4, 7]. One caveat of our study is that we focused only on sorafenib-induced ferroptosis. In addition to sorafenib inducing ferroptosis by inhibiting system Xc^−^ activity, regents such as RSL3 can also induce ferroptosis through GPX4 inactivation [30]. Whether Cdc25A is involved in other types of ferroptosis induced by other factors, including RSL3, remains unanswered [31]. Future studies are necessary to test this possibility.

PKM2, an isoform of pyruvate kinase, is a key enzyme for aerobic glycolysis and regulates the nonglycolytic transcription of many genes [19, 32, 33]. Metabolic reprogramming is an important feature of cancer cells and is critical for cancer cell survival and growth [34, 35]. Therefore, PKM2 is largely involved in cancer development. Its level is upregulated in a variety of human cancer cells, and this upregulation greatly contributes to cancer cell growth by reprogramming metabolism and activating the transcription of certain genes [19, 32, 36]. Regulation of the dephosphorylation of PKM2 has been shown to promote PKM2-dependent β-catenin transactivation, as well as cyclin D1 and c-Myc expression, resulting in an enhanced Warburg effect [23]. In our study, we found that Cdc25A suppressed sorafenib-induced ferroptosis and promoted cervical cancer cell growth by dephosphorylating PKM2 in the nucleus. Notably, the phosphorylation-mimicking mutant PKM2 S37D, but not PKM2-WT, reversed the effects of Cdc25A on autophagy and ferroptosis, indicating that the phosphorylation of PKM2 at Ser37, but not the total level of PKM2, matters. This result also highlights the importance of post-translational modifications of protein activity by kinases and phosphatases [37, 38]. In addition to the Ser37 site, there are many other phosphorylation residues on PKM2, such as tyrosine 105 and 83 and threonine 454 [20]. Furthermore, other types of post-translational modifications, such as acetylation and glycosylation, have been reported for PKM2 [20]. Distinct phosphorylation and post-translational modifications have different functions [20]. It might be interesting to examine the role of other modifications of PKM2 in cervical cancer.

As a member of the EGFP/ErbB family, ErbB2 is important for cell growth and proliferation [16, 39]. It is not surprising that dysregulated ErbB2 expression is implicated in various cancers [39]. Significantly, our results reveal an essential role of ErbB2 in ferroptosis. To the best of our knowledge, our study is the first to demonstrate this role. A previous study indicated that PKM2 can increase the transcription and translation of ErbB2 through pH3T11 and acetylation on histone H3 lysine 9 (H3K9Ac) [25]. We hypothesized that Cdc25A might induce similar signalling, and our aforementioned results support this model. More importantly, the knockdown of ErbB2 blocked the effects of Cdc25A overexpression on autophagy and ferroptosis, suggesting that ErbB2 is a crucial downstream player in this process. Notably, many molecules can modulate ErbB2 signalling, including mRNAs, and it might be interesting to examine whether these regulators have similar roles as Cdc25A in ferroptosis.

In summary, we demonstrated that the Cdc25A/PKM2/ErbB2 axis is a crucial pathway in sorafenib-induced ferroptosis of cervical cancer cells. Treatments targeting this pathway could be used or combined with sorafenib to combat cancer.

## Supplementary information

Supplementary figure legends

FIGS1

ORIGINAL EXPERIMENTAL (1)

ORIGINAL EXPERIMENTAL (2)

ORIGINAL EXPERIMENTAL (3)

ORIGINAL EXPERIMENTAL (4)

ORIGINAL EXPERIMENTAL (5)

## Data Availability

All data generated or analysed during this study are included in this published article.
