# Supplementary material for: Cdc25A inhibits autophagy-mediated ferroptosis by upregulating ErbB2 through PKM2 dephosphorylation in cervical cancer cells
Source: Cell Death Dis. 2021 Nov 6;12(11):1055. doi: 10.1038/s41419-021-04342-y (PMC8572225; doi:10.1038/s41419-021-04342-y)
Supplement: Supplementary file 1 — Supplementary figure legends [file 41419_2021_4342_MOESM1_ESM.docx]

**Supplementary figure legends**

**Supplemental Figure 1. Sorafenib induced autophagy-dependent ferroptosis.** (A) Relative cellular ROS levels in control cells or cells treated with sorafenib alone or sorafenib together with 3-MA. (B) Relative cellular iron levels in cells treated with sorafenib alone or sorafenib together with 3-MA. Each experiment was repeated three times. * *P* < 0.05; ** *P* < 0.01; *** *P* < 0.001.
