# Supplementary figures and images for: Cdc25A inhibits autophagy-mediated ferroptosis by upregulating ErbB2 through PKM2 dephosphorylation in cervical cancer cells
Source: Cell Death Dis. 2021 Nov 6;12(11):1055. doi: 10.1038/s41419-021-04342-y (PMC8572225; doi:10.1038/s41419-021-04342-y)

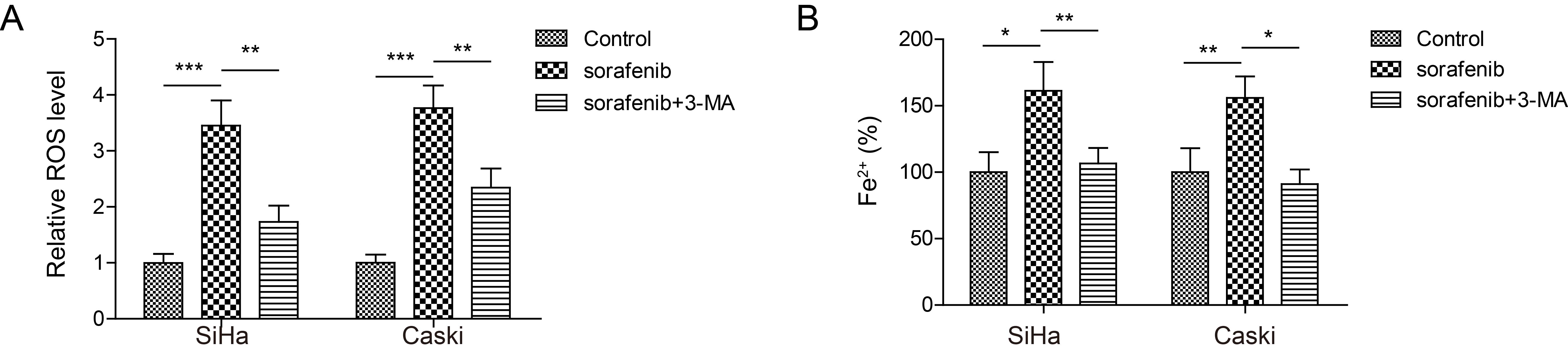

Supplement: Supplementary file 2 — FIGS1 [file 41419_2021_4342_MOESM2_ESM.jpg]

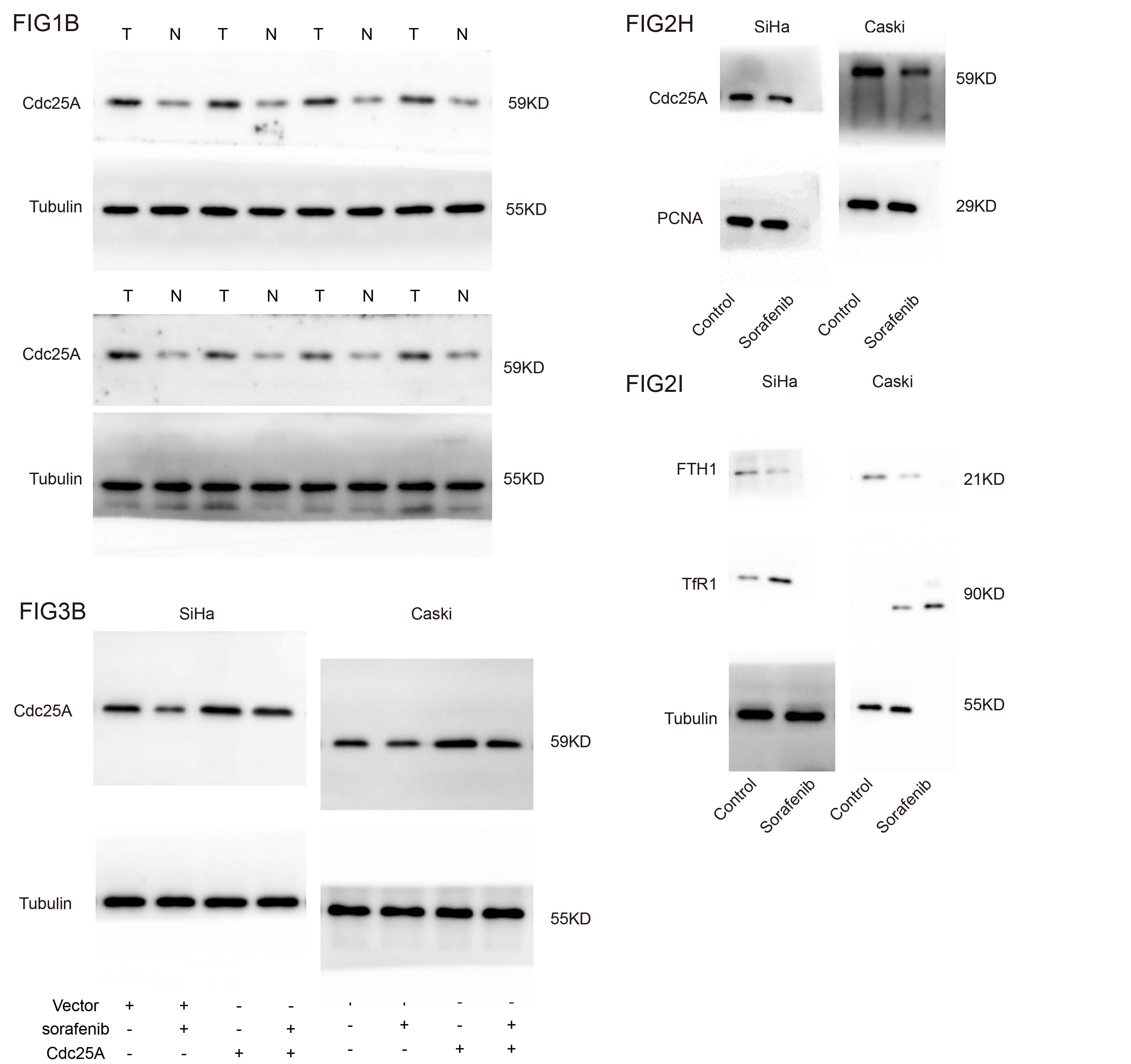

Supplement: Supplementary file 3 — ORIGINAL EXPERIMENTAL (1) [file 41419_2021_4342_MOESM3_ESM.jpg]

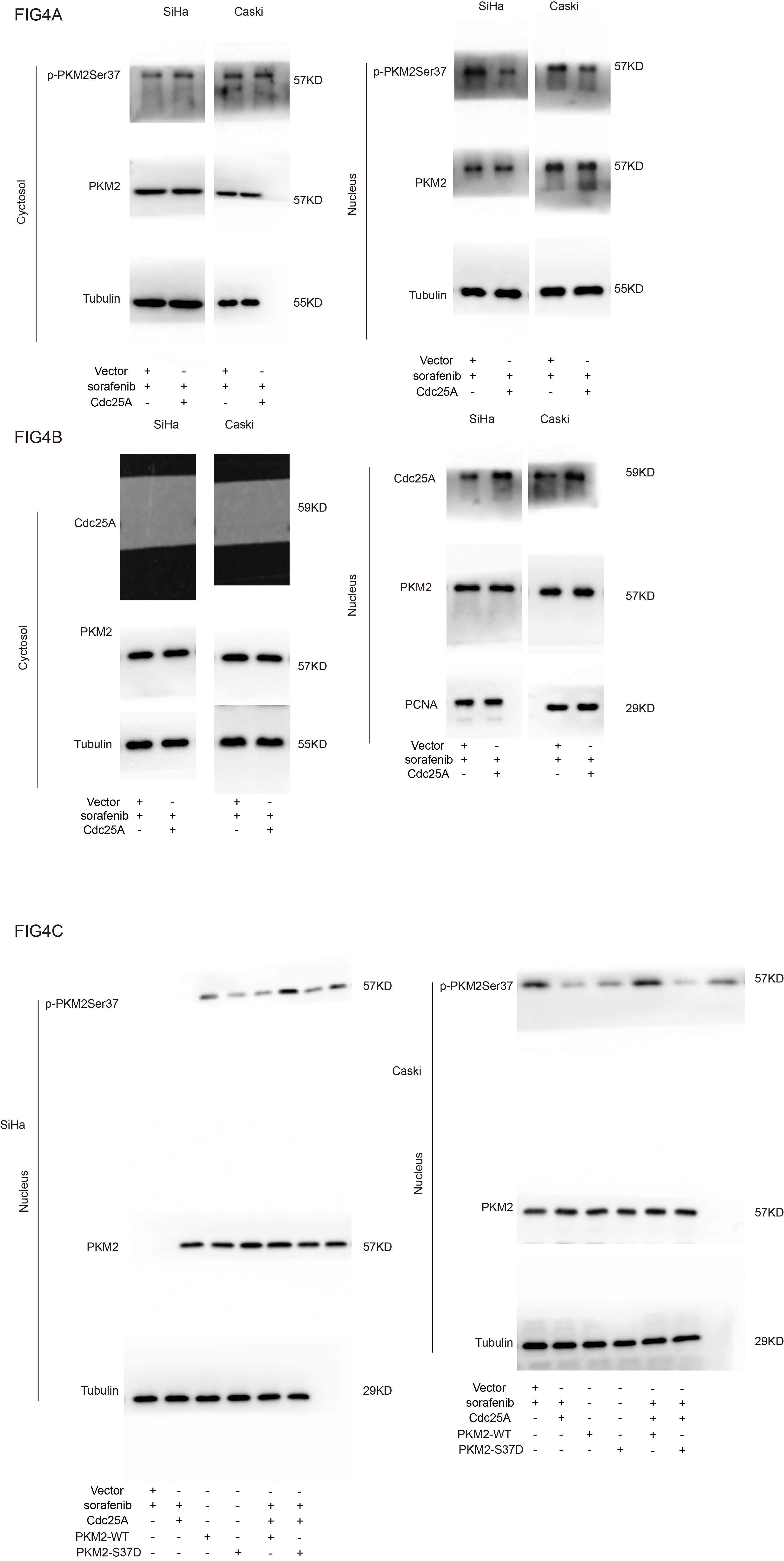

Supplement: Supplementary file 4 — ORIGINAL EXPERIMENTAL (2) [file 41419_2021_4342_MOESM4_ESM.jpg]

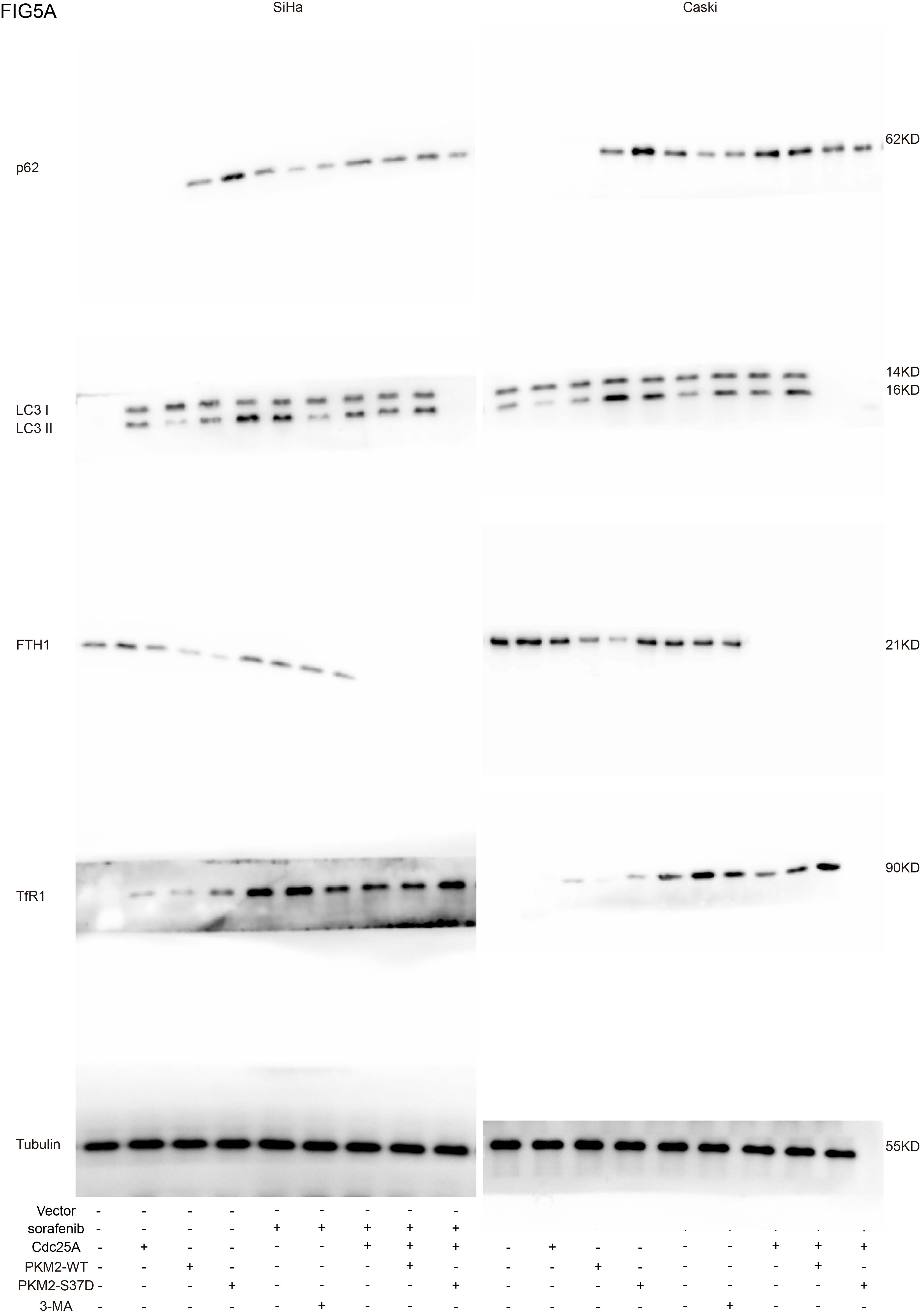

Supplement: Supplementary file 5 — ORIGINAL EXPERIMENTAL (3) [file 41419_2021_4342_MOESM5_ESM.jpg]

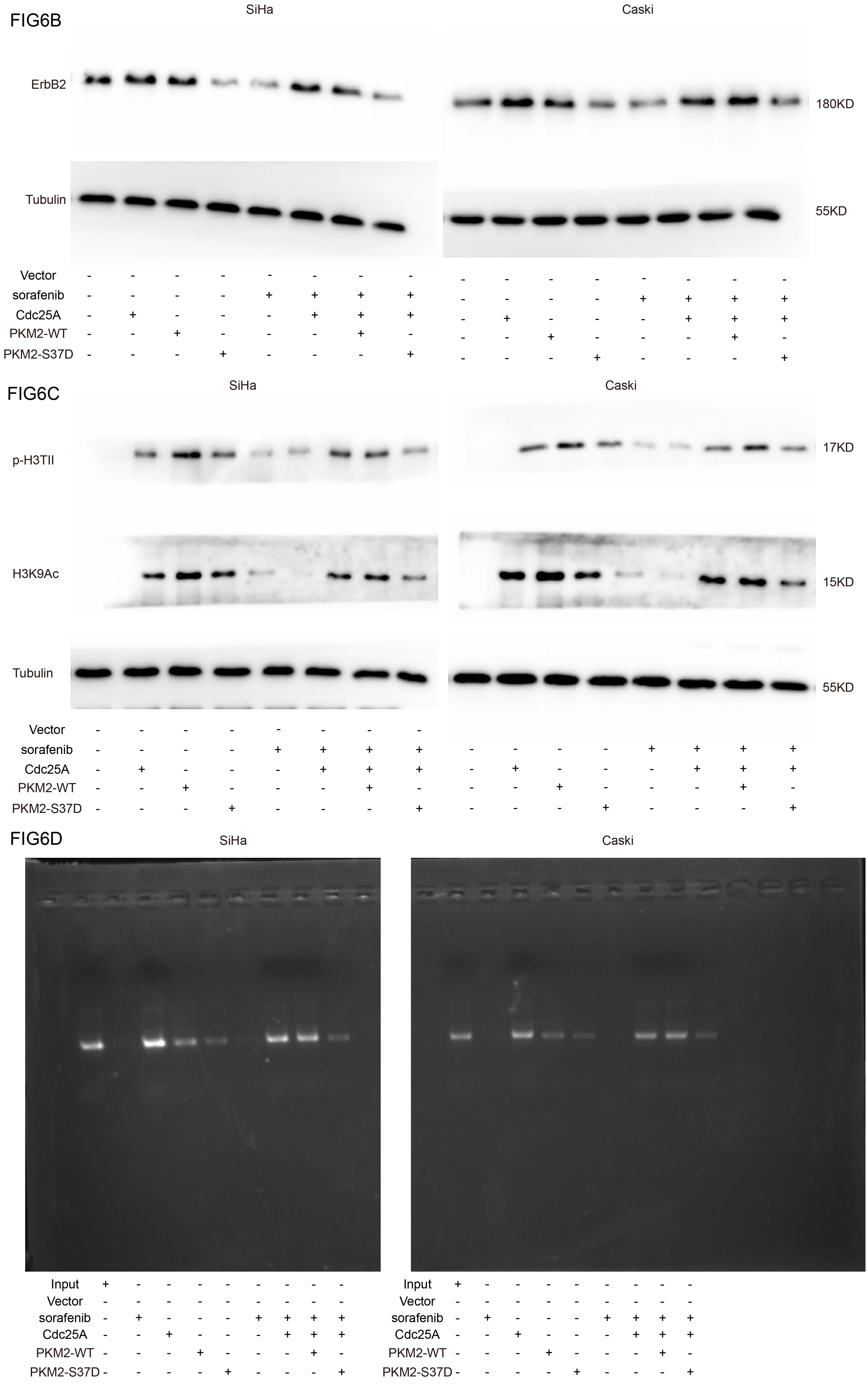

Supplement: Supplementary file 6 — ORIGINAL EXPERIMENTAL (4) [file 41419_2021_4342_MOESM6_ESM.jpg]

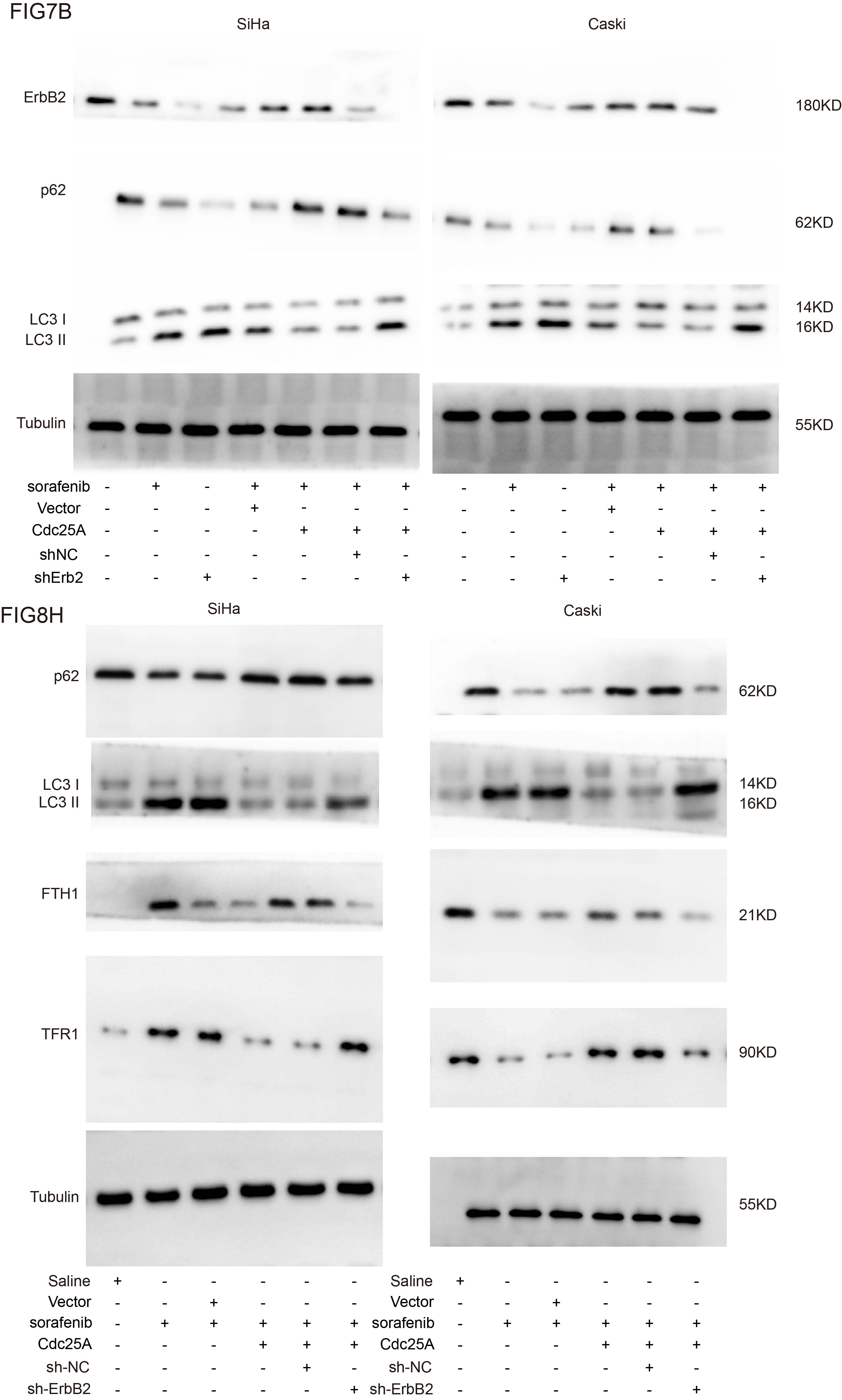

Supplement: Supplementary file 7 — ORIGINAL EXPERIMENTAL (5) [file 41419_2021_4342_MOESM7_ESM.jpg]
